# Supplementary material for: In vivo changes in zebrafish anesthetic sensitivity in response to the loss of kif5Aa are associated with the alteration of mitochondrial motility
Source: PLoS One. 2026 Jul 27;21(7):e0316959. doi: 10.1371/journal.pone.0316959 (PMC13405282; doi:10.1371/journal.pone.0316959)
Supplement: S1 Table — We assessed two genotypes, WT and kif5Aa KO neurons, and exposed them to 4 drugs with a no-drug (ND) control. The ND neurons were imaged in every experiment; hence the numbers were greater than that of the exposure groups. The “n” used and plotted as an open circle in Figs 4–6 and Supplemental Fig 6, was the total number of movies created. Within each movie, there was a range of traceable mitochondria, with a minimum allowable number of 5. The final column showed the total number of mitochondria traced per genotype per exposure group. (PDF) [file pone.0316959.s001.pdf]

	n = Number of movies	Range of mitochondria per movie	Total mitochondria traced
**WT neurons**			
No Drug	58	5 to 36	904
Propofol	31	6 to 23	416
Etomidate	25	7 to 31	394
Dexmedetomidine	30	9 to 36	632
Ketamine	30	8 to 44	485
***kif5Aa* KO neurons**			
No Drug	68	6 to 40	1280
Propofol	45	6 to 34	749
Etomidate	25	9 to 28	436
Dexmedetomidine	30	8 to 48	680
Ketamine	32	6 to 40	697

Supplemental Table 1. A summary of the number of mitochondria traced for the neuronal motility assays. We assessed two genotypes, WT and *kif5Aa* KO neurons, and exposed them to 4 drugs with a no-drug (ND) control. The ND neurons were imaged in every experiment; hence the numbers were greater than that of the exposure groups. The "n" used and plotted as an open circle in Figs 4-6 and Supplemental Fig 6, was the total number of movies created. Within each movie, there was a range of traceable mitochondria, with a minimum allowable number of 5. The final column showed the total number of mitochondria traced per genotype per exposure group.
