# Supplementary material for: In vivo changes in zebrafish anesthetic sensitivity in response to the loss of kif5Aa are associated with the alteration of mitochondrial motility
Source: PLoS One. 2026 Jul 27;21(7):e0316959. doi: 10.1371/journal.pone.0316959 (PMC13405282; doi:10.1371/journal.pone.0316959)
Supplement: S2 Fig — Movement of the 5 dpf WT sibling and kif5Aa KO larvae was assessed using the two behavioral assays, spontaneous (SM) and elicited movement (EM). A) The WT siblings demonstrated significantly less SM as compared to the kif5Aa KO larvae despite the loss of a swim bladder to maintain buoyancy. B) In contrast, when assessing EM, an acoustic startle response, there was a significant decrease in movement. The data was collected from the no-drug controls across all experiments using the 30-minute time point. The n = 40–41 with each point consisting of the average movement of 8–12 larvae. Error bars use standard deviation. Statistical significance using the Mann-Whitney test for nonparametric data. (PDF) [file pone.0316959.s003.pdf]

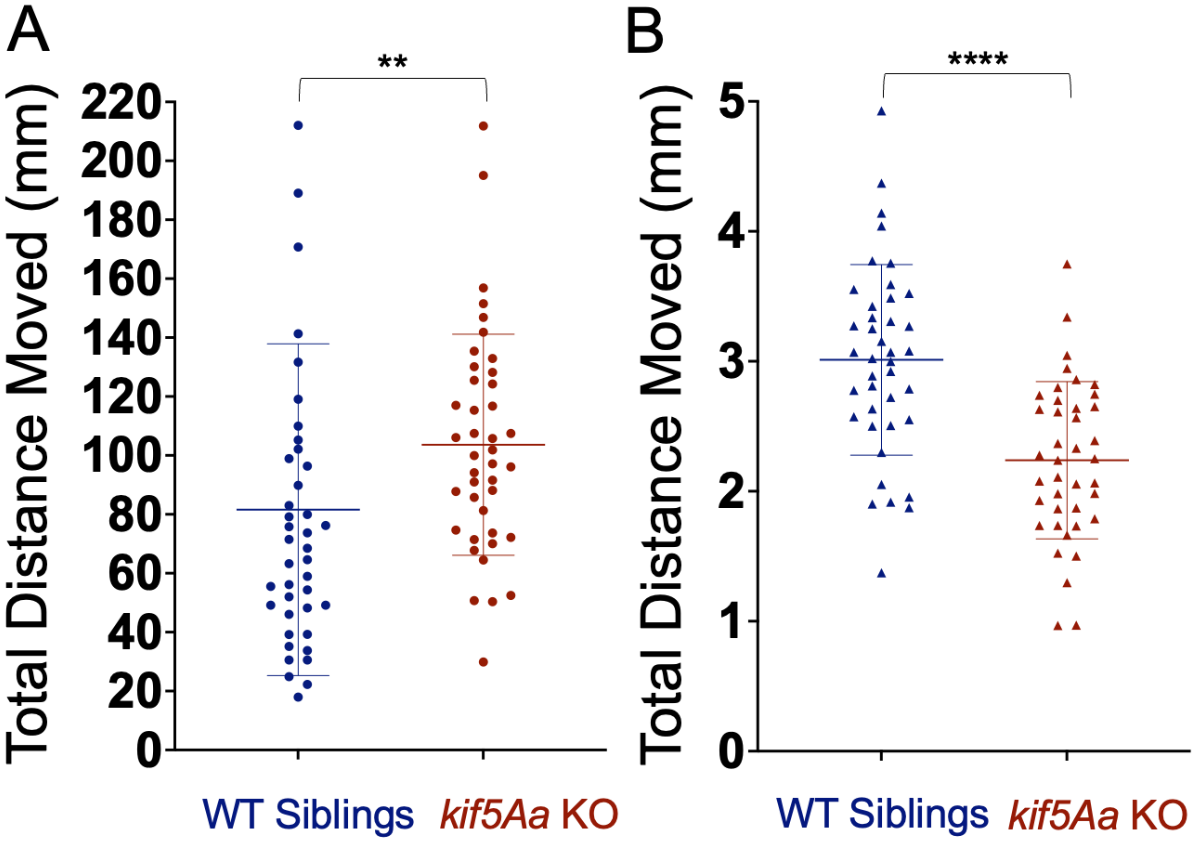

Supplemental Figure 2. Baseline movement was different in WT siblings versus the *kif5Aa* KO larvae. Movement of the 5 dpf WT sibling and *kif5Aa* KO larvae was assessed using the two behavioral assays, spontaneous (SM) and elicited movement (EM). A) The WT siblings demonstrated significantly less SM as compared to the *kif5Aa* KO larvae despite the loss of a swim bladder to maintain buoyancy. B) In contrast, when assessing EM, an acoustic startle response, there was a significant decrease in movement. The data was collected from the no-drug controls across all experiments using the 30-minute time point. The n=40-41 with each point consisting of the average movement of 8-12 larvae. Error bars use standard deviation. Statistical significance using the Mann-Whitney test for nonparametric data.
