# Supplementary material for: In vivo changes in zebrafish anesthetic sensitivity in response to the loss of kif5Aa are associated with the alteration of mitochondrial motility
Source: PLoS One. 2026 Jul 27;21(7):e0316959. doi: 10.1371/journal.pone.0316959 (PMC13405282; doi:10.1371/journal.pone.0316959)
Supplement: S3 Fig — The whole brains of kif5Aa KO larvae (B) and their WT siblings (A) were used to create primary cell cultures. A-B) Example of a bright field plate at 20x magnification. Due to the whole brain being used for cell culture, most of the neurons were surrounded by other nonneuronal cells. A’-A”’) Three example WT neurons overlaying brightfield and GFP showed the neuron cell boundary and the mitochondria within the cell, respectively. B’-B”’) Three example kif5Aa KO neurons, which demonstrated similar morphology to that of the WT siblings. (PDF) [file pone.0316959.s004.pdf]

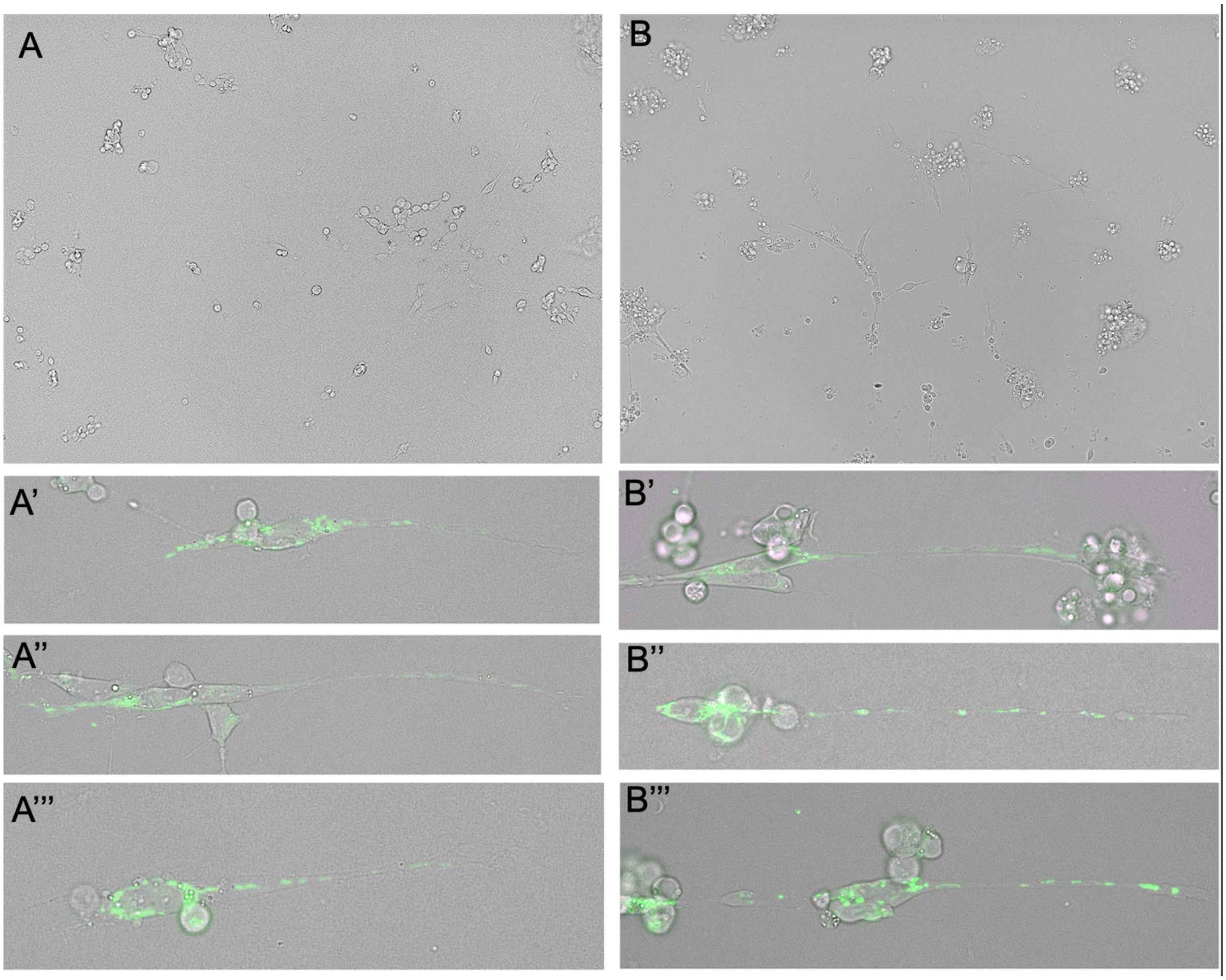

Supplemental Figure 3. Examples of WT and *kif5Aa* KO primary zebrafish neurons. The whole brains of *kif5Aa* KO larvae (B) and their WT siblings (A) were used to create primary cell cultures. A-B) Example of a bright field plate at 20x magnification. Due to the whole brain being used for cell culture, most of the neurons were surrounded by other nonneuronal cells. A'-A''') Three example WT neurons overlaying brightfield and GFP showed the neuron cell boundary and the mitochondria within the cell, respectively. B'-B''') Three example kif5Aa KO neurons, which demonstrated similar morphology to that of the WT siblings.
