# Supplementary material for: In vivo changes in zebrafish anesthetic sensitivity in response to the loss of kif5Aa are associated with the alteration of mitochondrial motility
Source: PLoS One. 2026 Jul 27;21(7):e0316959. doi: 10.1371/journal.pone.0316959 (PMC13405282; doi:10.1371/journal.pone.0316959)
Supplement: S4 Fig — The whole brains of kif5Aa KO larvae (B) and their WT siblings (A) were used to create primary cell cultures. A’-B’) The neurons were imaged every 3 seconds for 5 minutes, and the axons were traced in ImageJ. The movies of these axons were used to create kymographs. A”-B”) Kymographs were used to trace the mitochondria. Black is the background, and white shows mitochondrial fluorescence. Blue (A”) and red (B”) were a single example of tranced mitochondria. (PDF) [file pone.0316959.s005.pdf]

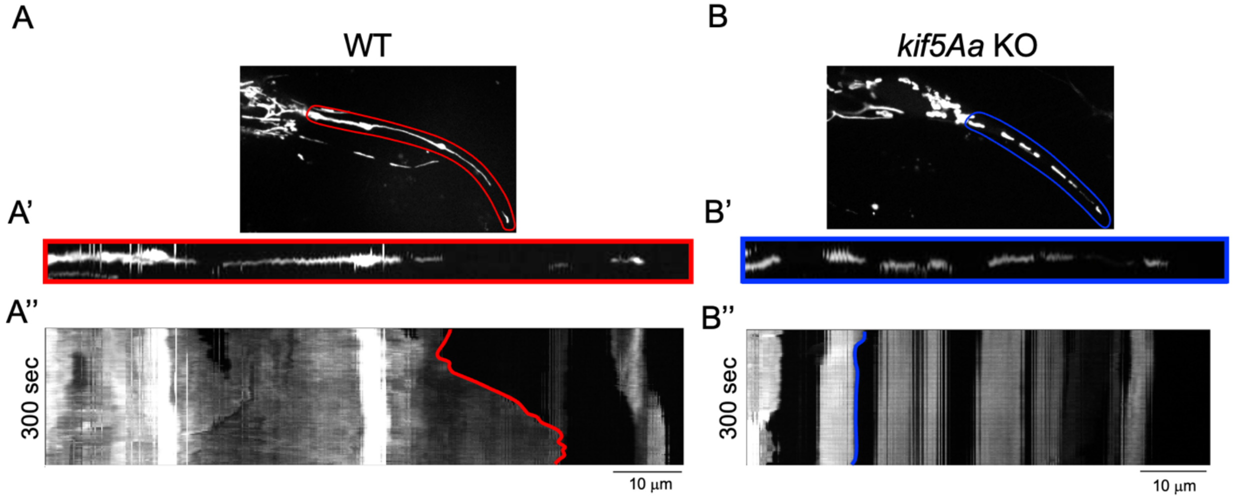

Supplemental Figure 4. WT and *kif5Aa* KO primary zebrafish neurons were used to measure mitochondrial motility. The whole brains of *kif5Aa* KO larvae (B) and their WT siblings (A) were used to create primary cell cultures. A'-B') The neurons were imaged every 3 seconds for 5 minutes, and the axons were traced in ImageJ. The movies of these axons were used to create kymographs. A''-B'') Kymographs were used to trace the mitochondria. Black is the background, and white shows mitochondrial fluorescence. Blue (A'') and red (B'') were a single example of tranced mitochondria.
