# Supplementary figures and images for: In vivo changes in zebrafish anesthetic sensitivity in response to the loss of kif5Aa are associated with the alteration of mitochondrial motility
Source: PLoS One. 2026 Jul 27;21(7):e0316959. doi: 10.1371/journal.pone.0316959 (PMC13405282; doi:10.1371/journal.pone.0316959)

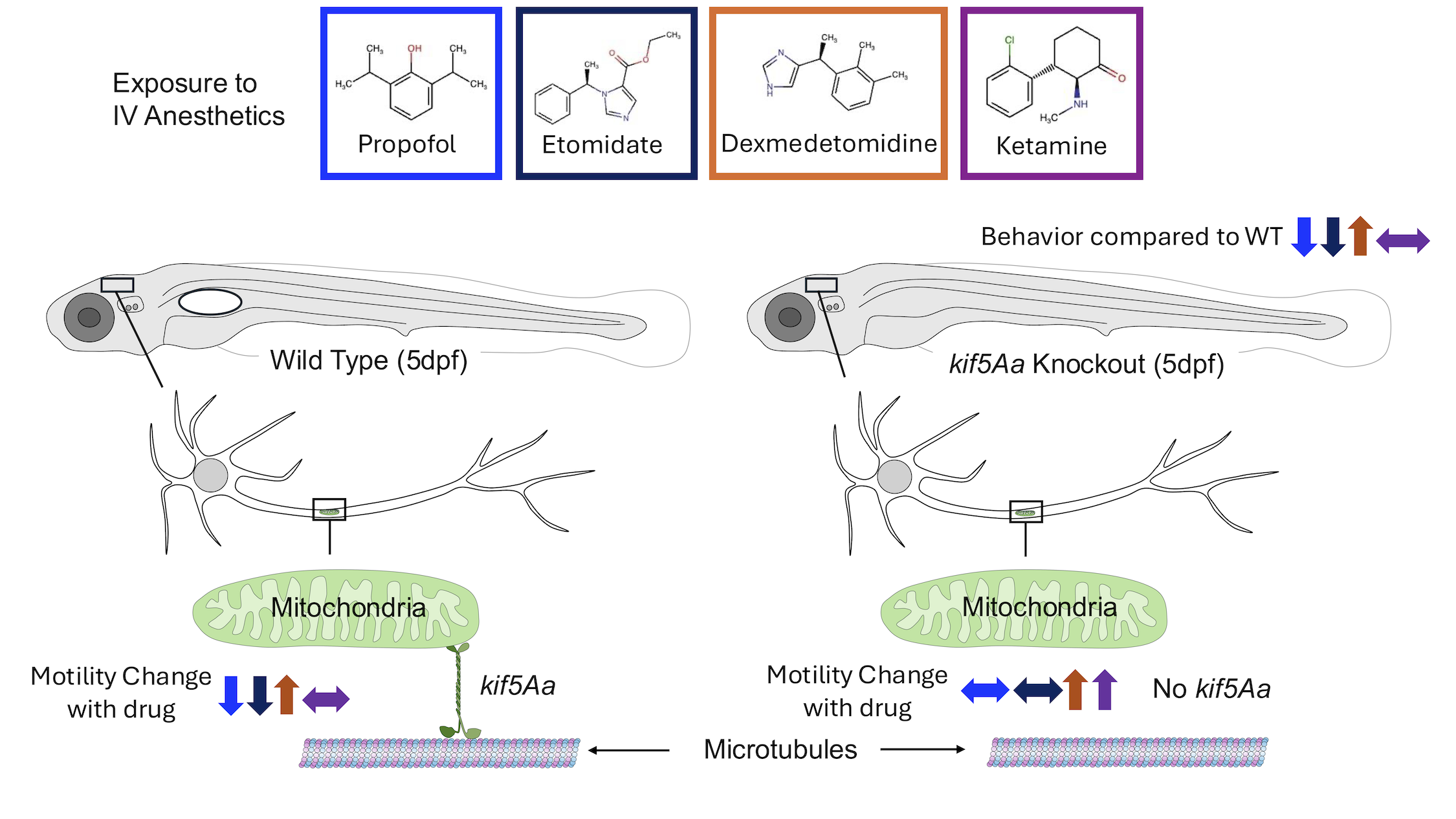

Supplement: S1 File — (TIFF) [file pone.0316959.s007.tiff]
