# Supplementary material for: In vivo changes in zebrafish anesthetic sensitivity in response to the loss of kif5Aa are associated with the alteration of mitochondrial motility
Source: PLoS One. 2026 Jul 27;21(7):e0316959. doi: 10.1371/journal.pone.0316959 (PMC13405282; doi:10.1371/journal.pone.0316959)
Supplement: S1 Fig — There is 67% homology between the paralogs Zebrafish kif5Aa and human kif5B. In the motor domain, the first approximately 340 amino acids, showed high sequence conservation (blue line). Additionally, the previously described amino acids directly binding propofol, leucine 258 and phenylalanine 318 (red boxes), were conserved. (PDF) [file pone.0316959.s002.pdf]

```
Kif5B (Homo sapiens) -MADLAECNIKVMCRFRPLNESEVNRGDKYIAKFQGEDTVVIASKPYAFDRVFQSSTSQE   59
Kif5Aa (Danio rerio) MTDAAAECNIKVLCRFRPLNQAEILRGDKFLPTFQGDDTVIIGGKSYAFDRVFPTNTTQE   60
                          *******:*******::*: ****:: .***:***:*..* ******* :.*:**

Kif5B (Homo sapiens) QVYNDCAKKIVKDVLEGYNGTIFAYGQTSSGKTHTMEGKLHDPEGMGIIPRIVQDIFNYI   119
Kif5Aa (Danio rerio) QVYNTCAKQIVKDVLGGYNGTIFAYGQTSSGKTHTMEGNLHDPQQMGIIPRIAEDIFNHI   120
                     **** ***:****** **********************:****: *******.:****:*

Kif5B (Homo sapiens) YSMDENLEFHIKVSYFEIYLDKIRDLLDVSKTNLSVHEDKNRVPYVKGCTERFVCSPDEV   179
Kif5Aa (Danio rerio) FSMDENLEFHIKVSYFEIYMEKIRDLLDVTKTNLSVHEDKNRVPYVKGCTERFVSSPEEV   180
                     :******************::********:************************.**:**

Kif5B (Homo sapiens) MDTIDEGKSNRHVAVTNMNEHSSRSHSIFLINVKQENTQTEQKLSGKLYLVDLAGSEKVS   239
Kif5Aa (Danio rerio) MDVIDEGKSNRHVAVTNMNEHSSRSHSIFLINIKQEHVETEQKLCGKLYLVDLAGSEKVS   240
                     **.*****************************:***:.:*****.***************

Kif5B (Homo sapiens) KTGAEGAVLDEAKNINKSLSALGNVISALAEGS-TYVPYRDSKMTRILQDSLGGNCRTTI   298
Kif5Aa (Danio rerio) KTGAAGAVLDEAKNINKSLSALGNVISALAEGTKSHVPYRDSKMTRILQDSLGGNCRTTM   300
                     **** ***************************: ::***********************:

Kif5B (Homo sapiens) VICCSPSSYNESETKSTLLFGQRAKTIKNTVCVNVELTAEQWKKKYEKEKEKNKILRNTI   358
Kif5Aa (Danio rerio) FICCSPSSYNDAETKSTLMFGQRAKTIKNTASINLELTAEQWKRKYEKEKEKNKSLRDSM   360
                     .*********::******:***********..:*:*********:********* **:::

Kif5B (Homo sapiens) QWLENELNRWRNGETVPIDEQFDKEKANLEAFTVDKDITLTND----------KPATAIG   408
Kif5Aa (Danio rerio) QRLELELRRWRNGEKVPETEQTDADVVKLDV---SEETSLDNEKSCERQRERERDSDTSS   417
                     * ** **.******.**  ** * : .:*:.   .:: :* *:          : : : .

Kif5B (Homo sapiens) VIGNFTDAERRKCEEEIAKLYKQLDDKDEEINQQSQLVEKLKTQMLDQEELLASTRRDQD   468
Kif5Aa (Danio rerio) IVVRISEEERQKYEEEIRKLYRQLDDKDDEINLQCQLVEKLKEQILDQDELLASTRGDSD   477
                     :: .::: **:* **** ***:******:*** *.******* *:***:******* *.*

Kif5B (Homo sapiens) NMQAELNRLQAENDASKEEVKEVLQALEELAVNYDQKSQEVEDKTKEYELLSDELNQKSA   528
Kif5Aa (Danio rerio) KVQTELGRLQTESESAKAEVREVLQALEELAVNYDQKSQEVEEKSLHNKQLAEQLSHKMA   537
                     ::*:**.***:*.:::* **:*********************:*: . : *:::*.:* *

Kif5B (Homo sapiens) TLASIDAELQKLKEMTNHQKKRAAEMMASLLKDLAEIGIAVGNNDVKQPE-GTGMIDEEF   587
Kif5Aa (Danio rerio) NLMELEAELAQMQEVSSQQRKRIADVLNGLMKDLSEFSSIVGNGELKLPVEISGAIEEEF   597
                     .* .::*** :::*::.:*:** *::: .*:***:*:.  ***.::* *   :* *:***

Kif5B (Homo sapiens) TVARLYISKMKSEVKTMVKRCKQLESTQTESNKKMEENEKELAACQLRISQHEAKIKSLT   647
Kif5Aa (Danio rerio) TVARLYISKIKSEVKSMVKRCRQLENMQLECHRKMEETSRELSSCQLLISQHEAKIRSLT   657
                     *********:*****:*****:***. * *.::****..:**::*** ********:***

Kif5B (Homo sapiens) EYLQNVEQKKRQLEESVDALSEELVQLRAQEKVH-EMEKEHLNKVQTANEVKQAVEQQIQ   706
Kif5Aa (Danio rerio) EYMQNVEQKKRQLEDNYDALSEELYTLQNSESHVAELDGGEK-ADREADGKKAA--VRPA   714
                     **:***********:. *******  *: .*.   *::  .    : *:  * *   :

Kif5B (Homo sapiens) SHRETHQKQISSLRDEVEAKAKLITDLQDQNQKMMLEQERLRVEHEKLKATDQEKSRKLH   766
Kif5Aa (Danio rerio) LHAESRQTQLSRLRDEINEKQRLIDELTDKNQALELELAHVRSSFSNLKLQDDSKSACLE   774
                      * *::*.*:* ****:: * :** :* *:** : **  ::* ...:**  *:.**  *.

Kif5B (Homo sapiens) ELTVMQDRREQARQDLKGLEETVAKELQTLHNLRKLFVQDLATRVKKSAEIDSDDTGGSA   826
Kif5Aa (Danio rerio) QMSFQQERHEQSKQDLKGLEETVARELQTLHNLRKLFVQDLTTRVKKSSEIGPDDSGGSN   834
                     :::. *:*:**::***********:****************:******:**. **:***

Kif5B (Homo sapiens) AQKQKISFLENNLEQLTKVHKQLVRDNADLRCELPKLEKRLRATAERVKALESALKEAKE   886
Kif5Aa (Danio rerio) TQKQKISFLENNLDQLTKVHKQLVRDNADLRCELPKLEKRLRSTAERVRALETALKDAKQ   894
                     :************:****************************:*****:***:***:**:

Kif5B (Homo sapiens) NASRDRKRYQQEVDRIKEAVRSKNMARRGHSAQIAKPIRPGQHPAASPTHPSAI-R----   941
Kif5Aa (Danio rerio) GAMNDRRRYQQEVERIRDAMRLRYPLRRPNAAQIAKPVRPGHHVTATSPTSFSVTRASKP   954
                     .* .**:******:**::*:* :   ** ::******:***:* :*:     :: *

Kif5B (Homo sapiens) ----GGGAFVQNS------------------------QPVAVRGGGGKQV----------   963
Kif5Aa (Danio rerio) ATSYSNARFLQDEKMENSQKTNTVGYSTVRSIDILSSCPLDVENGNSTDINDNRSDVHCG   1014
                         ... *:*:.                         *: *..*...::

Kif5B (Homo sapiens) ------------------- 963
Kif5Aa (Danio rerio) SMVDDASRVYIMQQETAAS 1033
```

Supplemental Figure 1. Zebrafish kif5Aa and human kif5B demonstrates homology within the motor domain. There is 67% homology between the paralogs Zebrafish kif5Aa and human kif5B. In the motor domain, the first approximately 340 amino acids, showed high sequence conservation (blue line). Additionally, the previously described amino acids directly binding propofol, leucine 258 and phenylalanine 318 (red boxes), were conserved.
